# Supplementary material for: Eco-Friendly and Economic, Adsorptive Removal of Cationic and Anionic Dyes by Bio-Based Karaya Gum—Chitosan Sponge
Source: Polymers (Basel). 2021 Jan 13;13(2):251. doi: 10.3390/polym13020251 (PMC7828559; doi:10.3390/polym13020251)
Supplement: Supplementary file 1 [file polymers-13-00251-s001.pdf]

Supplementary Materials

Eco-friendly and economic, adsorptive removal of cationic and anionic dyes by bio-based karaya gum - chitosan sponge

Rohith K. Ramakrishnan [1], Vinod V.T. Padil[1]*, Stanisław Wacławek[1], Miroslav Černík[1*], and Rajender S Varma [2]*

[1]Institute for Nanomaterials, Advanced Technologies and Innovation (CXI), Technical University of Liberec (TUL), Studentská 1402/2, Liberec 1, Czech Republic, 461 17; Telephone: +420 723372911.

[2]Regional Centre of Advanced Technologies and Materials, Palacký University in Olomouc, Šlechtitelů 27, 783 71 Olomouc, Czech Republic.

*Correspondence: Authors' Email: vinod.padil@tul.cz (V.V.T.P); Miroslav.cernik@tul.cz (M.C); and Varma.Rajender@epa.gov (R.S.V).

Number of pages: 4 (1 to 4)

Number of figures: 4

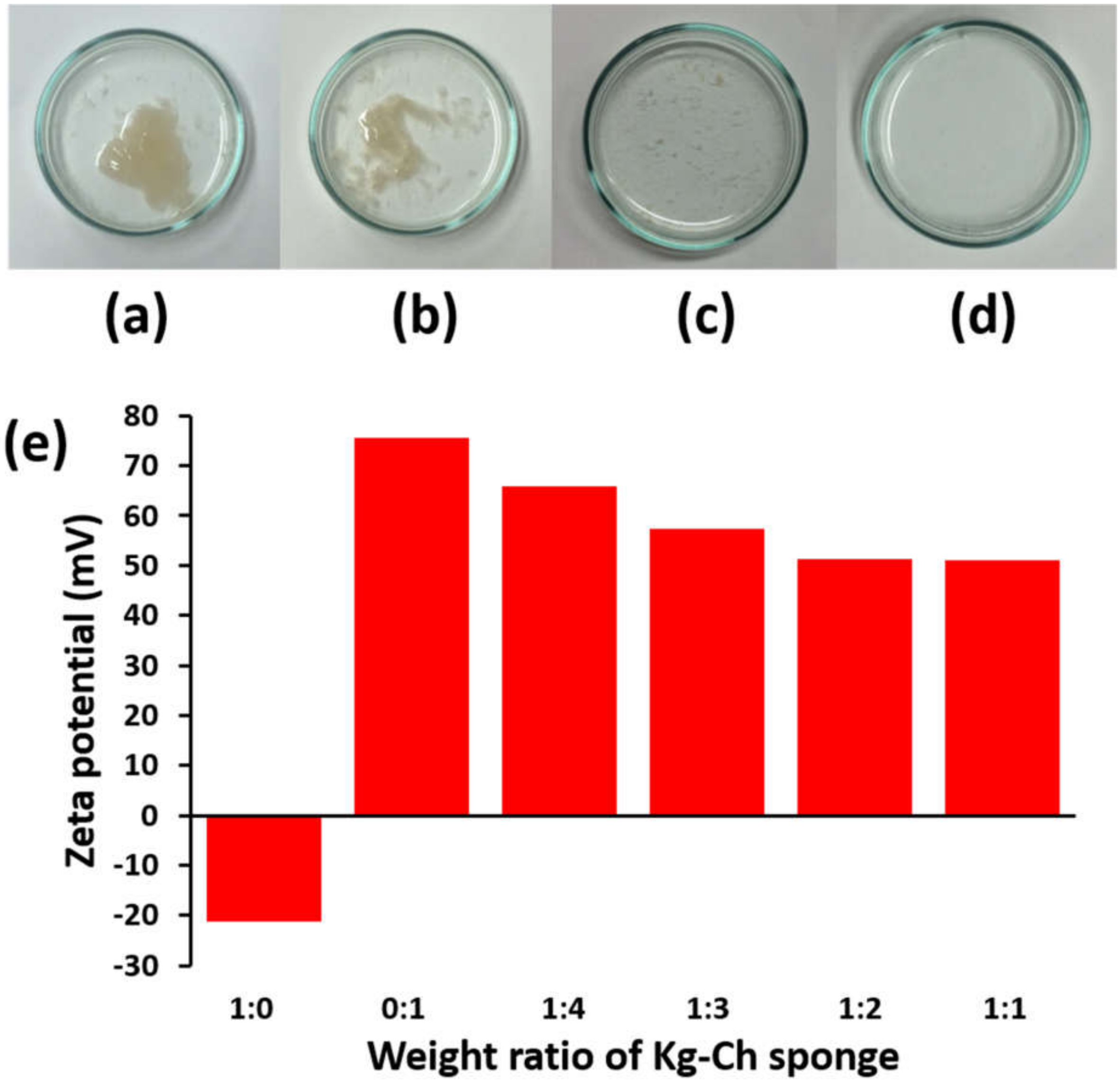

Figure S1. The ratio of Kg:Ch (a) 1:1 wt.%, (b) 1:2 wt.%, (c) 1:3 wt.% and (d) 1:4 wt.%; (e) Zeta potential values versus the weight ratios of Kg:Ch (1:0, 0:1; 1:4; 1:3; 1:2, and 1:1).

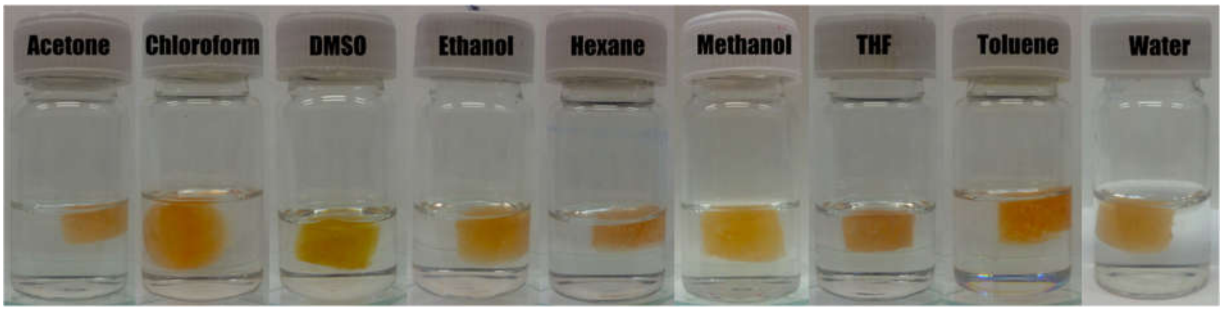

Figure S2. The chemical stability of Kg-Ch sponge immersed in various solvents for a period of more than 24 h at 25^0C.

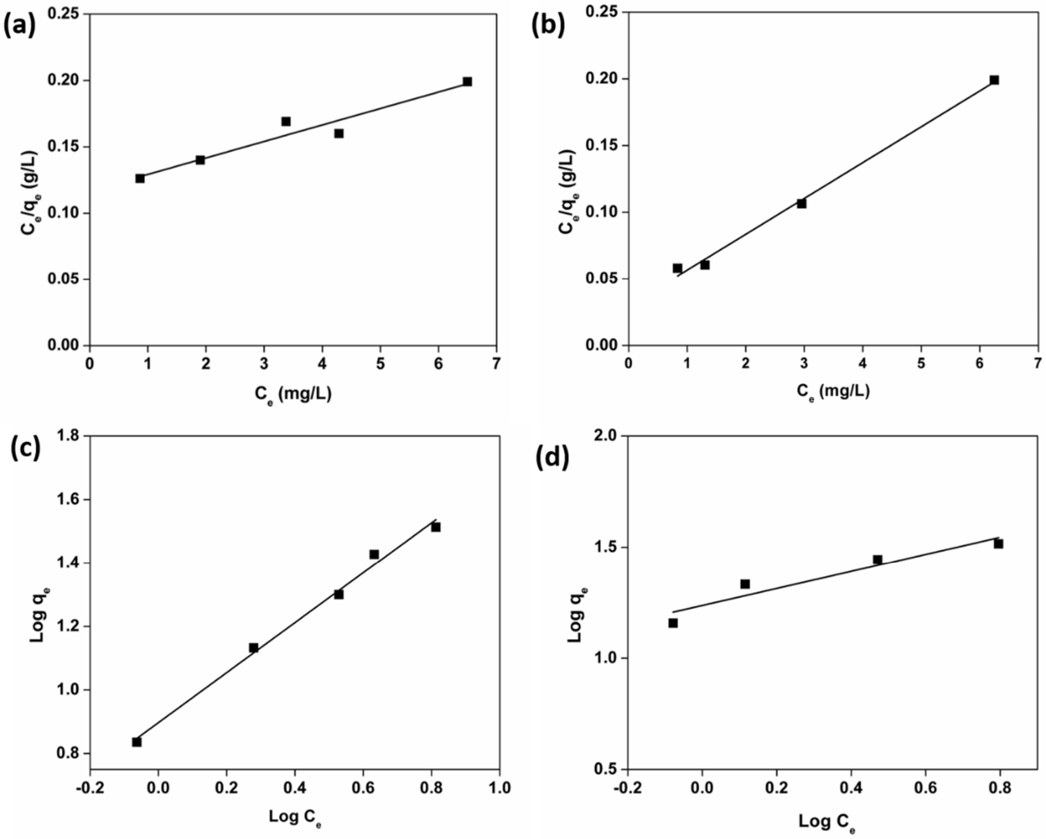

Figure S3: (a and b) Langmuir and (c and d) Freundlich adsorption isotherm models for the adsorption of MB and MO onto Kg-Ch sponge.

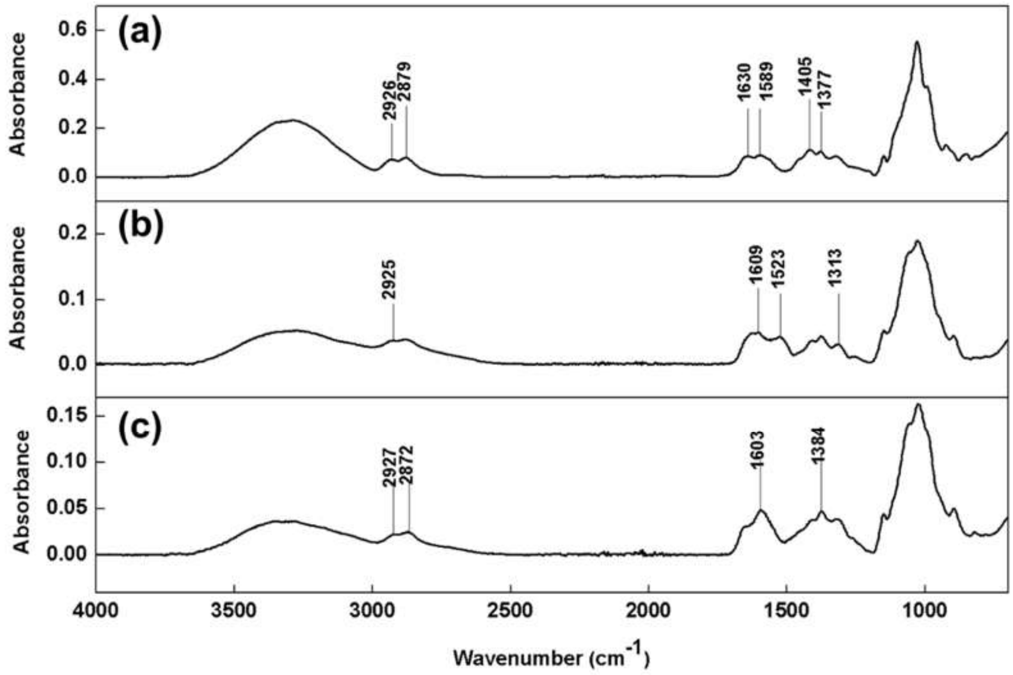

Figure S4. ATR-FTIR analysis of the (a) Kg-Ch sponge, (b) Kg-Ch sponge after MO adsorption and (c) Kg-Ch sponge after MB adsorption.
